# First Brazilian Symposium on Viruses of Microorganisms (BrVoM 2025)

**DOI:** 10.3390/v17121603

**Published:** 2025-12-11

**Authors:** Jônatas Santos Abrahão, Luiz Felipe Leomil Coelho, Amanda Stéphanie Arantes Witt, Ana Karoline da Nóbrega Nunes Alves, Anna Catarina Dias Soares Guimarães, Bárbara Stehling Ramos Silva, Bruna Nascimento Neiva, Bruno Fernandes de Oliveira, Jamile Dias, João Victor Rodrigues Pessoa Carvalho, Letícia Pereira Lopes, Matheus Felipe dos Reis Rodrigues, Matheus Gomes Barcelos, Nidia Esther Colquehuanca Arias, Poliane Zerbini, Vera Lucia dos Santos, Caio Ambrosio Leal-Dutra, Savio Torres de Farias, Rodrigo Araujo Lima Rodrigues, Juliana Reis Cortines, Otavio Henrique Thiemann, Paulo Boratto, Marcelo Henrique Aguiar de Freitas, Gabriel Magno de Freitas Almeida

**Affiliations:** 1 Department of Microbiology, Institute of Biological Sciences, Federal University of Minas Gerais, Belo Horizonte 31270-901, MG, Brazil; jonatas.abrahao@gmail.com (J.S.A.); asawitt1997@gmail.com (A.S.A.W.); anakarolinenunesalves@gmail.com (A.K.d.N.N.A.); annacatarinaanna@gmail.com (A.C.D.S.G.); basrs5775@gmail.com (B.S.R.S.); brunaneiva.ufmg@hotmail.com (B.N.N.); brunooliveira.ufmg@gmail.com (B.F.d.O.); jamiledias613@gmail.com (J.D.); jvrodrigues934@gmail.com (J.V.R.P.C.); leticiapl.bio@gmail.com (L.P.L.); matheusfelipe2552@gmail.com (M.F.d.R.R.); matheus.barcelos@outlook.com (M.G.B.); nidiaestherarias@gmail.com (N.E.C.A.); verabio@gmail.com (V.L.d.S.); rodriguesral07@gmail.com (R.A.L.R.); 2 Vaccine Laboratory, Department of Microbiology and Immunology, Institute of Biomedical Sciences, Federal University of Alfenas, Alfenas 37130-001, MG, Brazil; luiz.coelho@unifal-mg.edu.br; 3 Departamento de Microbiologia, Instituto de Biotecnologia Aplicada à Agropecuária (BIOAGRO), Universidade Federal de Viçosa, Viçosa 36570-000, MG, Brazil; palfenas@ufv.br; 4 Section for Ecology and Evolution, Department of Biology, University of Copenhagen, Universitetsparken 15, 2100 Copenhagen, Denmark; caio@bio.ku.dk; 5 Laboratory of Molecular and Computational Biology of Fungi, Institute of Biological Sciences, Federal University of Minas Gerais, Belo Horizonte 31270-901, MG, Brazil; 6 Laboratório de Genética Evolutiva Paulo Leminski, Departamento de Biologia Molecular, Universidade Federal da Paraíba, João Pessoa 58050-585, PB, Brazil; stfarias@yahoo.com.br; 7 Network of Researchers on the Chemical Evolution of Life (NoRCEL), Leeds LS7 3RB, UK; 8 Laboratório de Virologia e Espectrometria de Massas, Instituto de Microbiologia Paulo de Góes, Universidade Federal do Rio de Janeiro, Rio de Janeiro 21941-902, RJ, Brazil; cortines@micro.ufrj.br; 9 Physics Institute of São Carlos, University of São Paulo, Av. Trabalhador São Carlense 400, Sao Carlos 13566-590, SP, Brazil; thiemann@ifsc.usp.br; 10 Department of Genetics and Evolution, Federal University of São Carlos—UFSCar, São Carlos 13565-905, SP, Brazil; 11 Brazilian Biosciences National Laboratory—LNBio, Brazilian Center for Research in Energy and Materials—CNPEM, Campinas 13083-970, SP, Brazil; pvboratto@gmail.com; 12 Embrapa Genetic Resources & Biotechnology, Brasilia 70770-901, DF, Brazil; marcelo.freitas@embrapa.br; 13 Faculty of Biosciences, Fisheries and Economics, The Norwegian College of Fishery Science, UiT—The Arctic University of Norway, 9037 Tromsø, Norway

**Keywords:** bacteriophages, fungal viruses, giant viruses, microbial virology, Brazil, conference report

## Abstract

In recent decades, there has been an increased interest in viruses of microorganisms (VoM) and international efforts to gather researchers interested in them. Here, we describe the 1st Brazilian Symposium on Viruses of Microorganisms (BrVoM), held on 1 August 2025 at the Federal University of Minas Gerais (UFMG, Belo Horizonte, Brazil) with institutional support from the Federal University of Alfenas (UNIFAL) and the Brazilian Society for Virology (SBV). The symposium greatly surpassed expectations, gathering nearly 300 attendees from all Brazilian geographical regions. The scientific program included keynote and thematic lectures covering bacteriophages, fungal viruses, giant viruses, and microbial resources regulation. The event was remarkable for its collaborative spirit and inclusion of early career attendees. The success of this first edition highlights the vitality of the Brazilian community working on microbial viruses and sets the stage for future editions.

## 1. Introduction

Viruses are the most abundant organisms on Earth, found wherever life is present [1]. Most of the virosphere is composed of viruses that infect microbial life, collectively called viruses of microorganisms (VoM). This diverse viral group is composed of viruses of prokaryotes (phages) and viruses of unicellular eukaryotes (mycoviruses, large viruses of microalgae and giant viruses of protists) [2]. Although the discipline of virology has had an anthropocentric bias, in recent decades, the study of VoMs is increasingly gaining traction worldwide. Among the reasons are the importance of VoMs for the environment [3], for understanding microbial evolution [4], as tools for biotechnology [5] and for clinical purposes [6].

The VoM research field is broadly divided into two main focus areas, phages and viruses of unicellular eukaryotes. There has been an international effort into gathering virologists working with VoMs. The International Society for Viruses of Microorganisms (ISVM) has already organized eight international Viruses of Microbes conferences since 2010. These conferences took place in European countries, Georgia and more recently in Australia. Local VoM groups are being formed, such as the Belgian Society for Viruses of Microbes (BSVoM) founded in 2022 and the Danish Viruses of Microbes Network founded in 2025. However, besides the Africa Phage Forum, a collaborative network for phage research in Africa [7], and the Phages for Global Health [8] initiative, most of these networks are hosted and focused on the Global North.

South America has interesting ties to VoMs. From a historical and applied perspective, Brazil was a hotspot and reference for South American phage therapy between the 1920s to 1940s [9]. The efforts of Dr. Jose da Costa Cruz and the Oswaldo Cruz Institute at the time were so successful that they were even used as examples by Dr. George Eliava himself while asking for funds to expand his own Institute in Tbilisi in the 1930s [10]. After the gap in phage therapy created by the widespread use of antibiotics, phage research is growing in South America despite the lack of modern clinical cases. One exception is a single documented case of personalized phage therapy in Uruguay [11]. A recent systematic review show that Brazil leads the South American output of phage research, being responsible for 39% of the scientific publications in the region from 1989–2024 [12]. From an ecological perspective, the large biological diversity of Brazilian biomes is being shown to be true also to the Brazilian virosphere by efforts of The Giant Viruses Study Group [13], founded in 2011 at the Federal University of Minas Gerais [14]. Although Brazil has a strong virology community clustered within the Brazilian Society of Virology (SBV) [15] founded in 1986, traditionally the focus of the community has been on medically relevant viruses. Here, we report the initiative to gather Brazilian VoM researchers under the 1st Brazilian Symposium on Viruses of Microorganisms (BrVoM) in 2025. This successful event attracted a remarkable diversity of participants and consolidated a research community with similar interests and desire to keep the Brazilian VoM cluster alive in the upcoming years.

## 2. First Brazilian Symposium on Viruses of Microorganisms Organization

**Figure 1:**
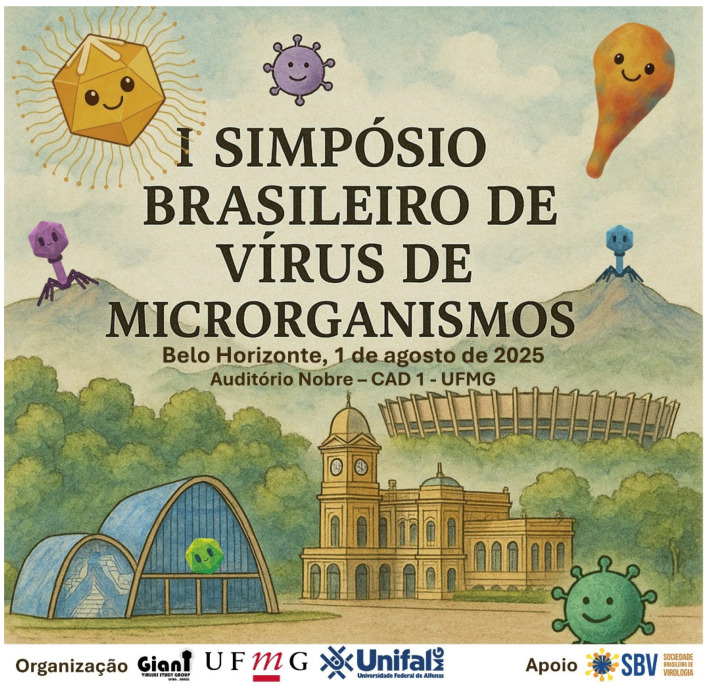
Flyer of the 1st Brazilian Symposium on Viruses of Microorganisms used for divulgating the event online and physically. Artificial intelligence was used to prepare this image (ChatGPT-5).

In the context described above, Brazilian virologists interested in VoMs organized the first edition of the Brazilian Symposium on Viruses of Microorganisms (BrVoM), held at the Federal University of Minas Gerais (UFMG), Belo Horizonte, with support from the Federal University of Alfenas (UNIFAL) and the Brazilian Society for Virology (SBV) (Figure 1). The event took place on 1 August 2025 from 9:30 to 17:30.

The organization committee opted to not charge any registration fee, making the event accessible to all and not excluding low-income students. The event language choice was Portuguese, another measure taken not to exclude any local participant. A small funding obtained from local biotech companies (Biocell/Molecular) was used to cover the preparation of participant tags, flyers and prizes given to the audience. These companies were present at the event in booths at the lobby to showcase their products and connect to the audience.

The event was advertised online, locally at the UFMG, and by the speakers within their own research networks. The local invited speakers participated on a voluntary basis, covering their own expenses, which underscores the strong sense of collaboration and commitment in this scientific field. The speakers were chosen based on their contributions to the VoM field and were all established early or mid-career researchers. The agenda for the event is shown in Table 1.

The event exceeded all expectations: initially planned for a modest audience of 50 participants, it ultimately brought together almost 300 researchers, students, professionals and even participants from the general public, making it one of the most significant virology events in Brazil in 2025. The participants who registered for the event represented 15 Brazilian states, covering all five regions of the Country, and were associated with 51 different Brazilian institutions. A large proportion of early career attendees was noted, pointing out that the VoM field will have a long impact in the next generation of Brazilian virologists.

## 3. First Brazilian Symposium on Viruses of Microorganisms Report

### 3.1. The Start of the Event

The meeting was opened with a round table composed of representants from the organizing committee (Professor Jonatas dos Santos Abrahao from UFMG and Professor Luiz Felipe Leomil Coelho from UNIFAL), the head of the Microbiology Department of the UFMG (Professor Daniel Assis Santos) and Professor Betania Paiva Drummond representing the Brazilian Society for Virology.

The first talk was a keynote lecture by associate Professor Gabriel Magno de Freitas Almeida (Arctic University of Norway), entitled One Hundred Years of Phage Therapy and the Revival of Its Prophylactic Character in Contemporary Times. His presentation revisited the historical development of phage therapy, including the role Brazil had in the first half of the 20th century [9], ending with the perspective of applying phage–bacteria–mucin dynamics [16,17] as a mean to revive a prophylactic approach to phage use in the present. He mentioned the lack of opportunities to start phage projects while an early career researcher in Brazil a decade ago, which made him go abroad to pursue it.

### 3.2. Bacteriophage Biology Session

Professor Luiz Felipe Leomil Coelho (UNIFAL-MG) discussed Phage-based strategies to control *Pseudomonas aeruginosa*, presenting results from phages isolated by his research group which were tested in vivo with a rational prophylactic approach based on mucosal dynamics [18]. Two similar Brazilian phages were compared, showing that phage VAC3 demonstrated superior replication in *P. aeruginosa* exposed to mucin in vitro and showed a stronger retention within the respiratory tract of C57BL/6 mice. Importantly, pre-exposure to VAC3 protected mice from an otherwise lethal challenge with *P. aeruginosa*, whereas phage VAC1 failed to confer such protection. These findings highlight that phages adapted to mucosal environments hold potential as prophylactic agents against ESKAPE pathogens.

Professor Poliane Zerbini (Federal University of Viçosa, UFV) provided an overview of ongoing efforts to characterize the natural diversity and evolutionary dynamics of phages infecting Ralstonia species, responsible for bacterial wilt, an important constraint to global crop production. Her group has been uncovering how phage–host interactions influence the population structure, adaptability, and persistence of *Ralstonia* species in diverse environments, contributing to a broader understanding of viral roles in shaping the ecology of phytopathogenic bacteria and in driving the coevolutionary processes that define host–virus relationships in the soil microbiome. In addition, she emphasized the translational potential of this research for sustainable agriculture using phage-based biocontrol formulations aimed at suppressing *Ralstonia* populations in the field, bridging fundamental and applied virology.

Professor Vera Lúcia dos Santos (UFMG) delivered a lecture on the use of phages as sustainable alternatives for controlling biofilms in industrial systems, with a particular focus on cooling water systems. She addressed the operational and environmental challenges associated with biofilm formation, including corrosion of infrastructure, reduced heat exchange efficiency, increased reliance on chemical biocides, and the emergence of antimicrobial-resistant communities. Complementing the molecular and microbiological data, Dr. Santos presented results from controlled pilot-scale reactor systems that reproduce industrial conditions. By integrating microbial ecology, biotechnology, and process engineering, her work positions phage-based interventions as promising, scalable, and environmentally responsible alternatives to conventional chemical control approaches.

### 3.3. Fungal Viruses Session

Dr. Caio Leal-Dutra (UFMG/University of Copenhagen, Denmark) presented Silent Viruses in a Symbiosis That Challenges Time, exploring the ecological relevance of fungal viruses and their impact on symbiotic systems inspired by the classic discovery of mycoviruses in Yellowstone endophytic fungi [19] and by evidence of viral sequences hidden in fungal transcriptomes [20]. Combining transmission electron microscopy with next-generation sequencing, the team characterized two novel +ssRNA mycoviruses cohabiting the domesticated fungus of leafcutter ants, Leucoagaricus gongylophorus tymo-like virus 1 (LgTlV1) and Leucoagaricus gongylophorus magoulivirus 1 (LgMV1) [21]. He concluded by discussing ongoing efforts to understand how these viruses shape the stability of one of nature’s most remarkable symbioses, potentially acting as neutral passengers, detrimental agents, or even hidden mutualists.

### 3.4. Discovery and Characterization of Viruses of Unicellular Eukaryotes Session

The afternoon sessions were dedicated to giant viruses. Professor Jônatas Abrahão (UFMG) spoke about Brazilian contributions to the study of giant amoebal viruses. The talk focused on the biology and evolution of giant amoeba-infecting viruses, highlighting the research conducted by his group in Brazil since 2011. Among the most impactful results, he mentioned the discovery of Tupanvirus [22] and Yaravirus [23], two unique representatives that have significantly expanded our understanding of the diversity and biological peculiarities of these microorganisms. He also emphasized how the contributions of the international scientific community have been transforming the perception of the limits of the virosphere. In this context, studies on giant viruses not only challenge traditional concepts in virology but also raise new questions about the origin, evolution, and ecological roles of these viruses, strengthening the relevance of this emerging field of research.

Professor Sávio Torres (UFPB) discussed Recent Insights into Yaravirus biology. Intrigued by the lack of similarity Yaravirus proteins have to other deposited sequences, his group decided to reanalyze the proteome of Yaravirus in an attempt to uncover possible clues about the functions encoded in its genome. By using a similarity search based on three-dimensional structures and not on primary amino acid sequences, allowing to match the modelled proteins from Yaravirus to matches with known function. It was proposed that Yaravirus exhibits a modular genomic organization, in which different genes encode parts of a protein, which are then reorganized at the protein level to restore full functionality. Many proteins were matched to functions related to the Krebs cycle and energy metabolism, highlighting that idiosyncrasies of Yaravirus lie not only in its size or mysterious genome but also in the unique ways its genome functions.

Professor Rodrigo Rodrigues (UFMG) concluded the session by discussing the hidden diversity of microalgal viruses in continental waters. Among the known algal viruses, the chloroviruses are the most studied, having large dsDNA viruses and infecting *Chlorella*-like algae [24]. His presentation highlighted the importance of isolating and genomically characterizing chloroviruses from new environments. These works have enabled the establishment of classification criteria for chloroviruses, revealing the existence of 20 viral species grouped into three subgenera based on multiple lines of evidence, including host range, phylogeny, and comparative genomics [25,26,27]. The search for new chloroviruses in Brazilian biomes led to the isolation and characterization of the first microalgal viruses in Brazil, opening new avenues for discovery and advances in the field of aquatic giant viruses.

### 3.5. Giant Viruses—Host Interactions Session

Professor Juliana Cortines (Federal University of Rio de Janeiro, UFRJ/University of Connecticut, USA) spoke about the Role of Transition Metals in the Replication of Mimiviruses. She outlined a continuum of studies beginning with cryo-electron microscopy of mimiviruses and proteomic analysis of the viral components released inside their Acanthamoeba hosts upon infection [28]. Among the identified proteins, those with metal-binding capacity were particularly significant, prompting further investigation into the role of metals during mimivirus infection [29]. Building on this, she presented evidence that iron acts as an enhancer of infectivity: intracellular iron levels increase as infection progresses, and viral titers rise in the presence of supplemental iron [30].

Professor Otavio Thiemann (University of São Paulo, USP) then presented his findings on Niemeyer virus viral factories using X-ray imaging techniques. He used synchrotron beamlines to investigate the structure of the viral factories (VF) of amoeba infected by Niemeyervirus. Soft X-ray Tomography (Cryo-SXT) of the Mistral beamline (ALBA synchrotron, Spain) revealed the formation of immature and mature capsids from the internal region of the VF to the exterior. Nanotomography Ptychography (CXDI) experiments at the Cateretê beamline (SIRIUS, Brazil) highlighted significant structural changes during the infection and viral biosynthesis in its native three-dimensional form. These techniques contribute to a better understanding of the role of the viral factory and the changes in host cell architecture during infection, with minimal interference with the native structure of the cells and target complexes.

Dr. Paulo Boratto (Brazilian Center for Research in Energy and Materials, CNPEM) discussed amoeba chemical signaling to resist giant virus infections. He used the long-standing host–parasite relationship between amoebae and giant viruses as a framework to investigate ancestral immune strategies in eukaryotes. By employing amoebozoan species and some *Nucleocytoviricota* viruses as model representatives of ancient hosts and pathogens, he demonstrated that viral infection triggers the amoebae to release extracellular alarm signals, which induce neighboring cells to rapidly undergo encystment and thereby establish resistance against giant virus infection.

### 3.6. Event Closure

The symposium concluded with a closing lecture by Dr. Marcelo Henrique Aguiar de Freitas (Embrapa), entitled Deposit of Microorganisms in the Embrapa Genetic Bank and International Instruments. He addressed the regulatory and biosafety aspects related to the deposition of microbial strains in national and international collections, a highly relevant topic for researchers working with VoMs. He introduced the Germplasm Curation System [31] and the Curation of Microorganisms [32] from Embrapa, which contains 10 large biological collections with different thematic axes and more than 15 associated biological collections, which has been improving to increase the provision of services to society, such as: the use of the Alelo Platform for managing RGs, the deposit of biological material of microorganisms and the implementation of an International Depository Authority (IDA) to comply with the Budapest Treaty.

The event ended with acknowledgments to the participating institutions, speakers, students, and the organizing committee. A prize draw was made to give books, coffee mugs and memberships to the Brazilian Society of Virology to the audience. Feedback from the speakers and audience is being positive, and plans for a follow-up meeting are being made.

## 4. Conclusions

The 1st Brazilian Symposium on Viruses of Microorganisms was a landmark event for the Brazilian virology community. By bringing together nearly 300 participants in a free and fully collaborative initiative, the symposium demonstrated the growing relevance of microbial virology in Brazil and created a new platform for scientific discussion, networking, and training. The breadth of topics covered—from bacteriophages to giant viruses—reflects the diversity and strength of research in the country. The overwhelming success of this first edition sets the stage for the BrVoM to become a recurring scientific meeting, fostering further integration of the Brazilian and international communities dedicated to the study of viruses of microorganisms.

## Figures and Tables

**Table 1:** Agenda of the 1st Brazilian Symposium on Viruses of Microorganisms.

Time	Session/Topic	Speaker(s)	Institution	Title/Subject
Morning	Opening Keynote	Prof. Gabriel M.F. Almeida	Arctic University of Norway	One Hundred Years of Phage Therapy and the Revival of Its Prophylactic Character in Contemporary Times
	Bacteriophage Biology	Prof. Luiz Felipe L. Coelho	UNIFAL-MG	Phage-based strategies to control *Pseudomonas aeruginosa*
		Prof. Poliane Zerbini	UFV	Ecology and evolution of bacteriophages infecting *Ralstonia* species and their potential use in biocontrol
		Prof. Vera Lúcia dos Santos	UFMG	Use of phages as sustainable alternatives for controlling biofilms in industrial systems
	Fungal Viruses	Dr. Caio Leal Dutra	UFMG/University of Copenhagen	Silent Viruses in a Symbiosis That Challenges Time
Afternoon	Discovery and Characterization of Viruses of Unicellular Eukaryotes (Chair: Prof. Jônatas Abrahão, UFMG)	Prof. Jônatas Abrahão	UFMG	Brazilian contributions to the study of giant amoebal viruses
		Prof. Sávio Torres de Farias	UFPB	Recent insights into *Yaravirus* biology
		Prof. Rodrigo A. L. Rodrigues	UFMG	Hidden diversity of algal viruses in continental waters
	Giant Viruses—Host Interactions (Chair: Prof. Juliana Cortines, UFRJ/University of Connecticut)	Prof. Juliana R. Cortines	UFRJ/University of Connecticut	Role of transition metals in the replication of mimiviruses
		Prof. Otavio H. Thiemann	USP	Observation of the *Niemeyer* virus viral factory through X-ray imaging techniques
		Dr. Paulo Boratto	CNPEM	Amoeba chemical signaling to resist giant virus infections
Closing	Closing Lecture	Dr. Marcelo Henrique Aguiar de Freitas	Embrapa	Deposit of Microorganisms in the Embrapa Genetic Bank and International Instruments
